# Artificial intelligence for differentiating COVID-19 from other viral pneumonias on CT: comparative analysis of different models based on quantitative and radiomic approaches

**DOI:** 10.1186/s41747-022-00317-6

**Published:** 2023-01-24

**Authors:** Giulia Zorzi, Luca Berta, Francesco Rizzetto, Cristina De Mattia, Marco Maria Jacopo Felisi, Stefano Carrazza, Silvia Nerini Molteni, Chiara Vismara, Francesco Scaglione, Angelo Vanzulli, Alberto Torresin, Paola Enrica Colombo

**Affiliations:** 1 Postgraduate School of Medical Physics, Università degli Studi di Milano, via Giovanni Celoria 16, 20133 Milan, Italy; 2 Department of Medical Physics, ASST Grande Ospedale Metropolitano Niguarda, Piazza Ospedale Maggiore 3, 20162 Milan, Italy; 3 Department of Physics, INFN Sezione di Milano, via Giovanni Celoria 16, 20133 Milan, Italy; 4 Postgraduate School of Diagnostic and Interventional Radiology, Università degli Studi di Milano, via Festa del Perdono 7, 20122 Milan, Italy; 5 Department of Radiology, ASST Grande Ospedale Metropolitano Niguarda, Piazza Ospedale Maggiore 3, 20162 Milan, Italy; 6 Department of Physics, Università degli Studi di Milano, via Giovanni Celoria 16, 20133 Milan, Italy; 7 Chemical-Clinical and Microbiological Analyses, ASST Grande Ospedale Metropolitano Niguarda, Milan, Italy; 8 Department of Oncology and Hemato-Oncology, Università degli Studi di Milano, via Festa del Perdono 7, 20122 Milan, Italy

**Keywords:** Artificial intelligence, COVID-19, Lung, Radiomics, Tomography (x-ray, computed)

## Abstract

**Background:**

To develop a pipeline for automatic extraction of quantitative metrics and radiomic features from lung computed tomography (CT) and develop artificial intelligence (AI) models supporting differential diagnosis between coronavirus disease 2019 (COVID-19) and other viral pneumonia (non-COVID-19).

**Methods:**

Chest CT of 1,031 patients (811 for model building; 220 as independent validation set (IVS) with positive swab for severe acute respiratory syndrome coronavirus-2 (647 COVID-19) or other respiratory viruses (384 non-COVID-19) were segmented automatically. A Gaussian model, based on the HU histogram distribution describing well-aerated and ill portions, was optimised to calculate quantitative metrics (QM, *n* = 20) in both lungs (2L) and four geometrical subdivisions (GS) (upper front, lower front, upper dorsal, lower dorsal; *n* = 80). Radiomic features (RF) of first (RF1, *n* = 18) and second (RF2, *n* = 120) order were extracted from 2L using PyRadiomics tool. Extracted metrics were used to develop four multilayer-perceptron classifiers, built with different combinations of QM and RF: *Model1* (RF1-2L); Model2 (QM-2L, QM-GS); Model3 (RF1-2L, RF2-2L); Model4 (RF1-2L, QM-2L, GS-2L, RF2-2L).

**Results:**

The classifiers showed accuracy from 0.71 to 0.80 and area under the receiving operating characteristic curve (AUC) from 0.77 to 0.87 in differentiating COVID-19 *versus* non-COVID-19 pneumonia. Best results were associated with *Model3* (*AUC* 0.867 ± 0.008) and *Model4* (*AUC* 0.870 ± 0.011. For the IVS, the AUC values were 0.834 ± 0.008 for *Model3* and 0.828 ± 0.011 for *Model4*.

**Conclusions:**

Four AI-based models for classifying patients as COVID-19 or non-COVID-19 viral pneumonia showed good diagnostic performances that could support clinical decisions.

**Supplementary Information:**

The online version contains supplementary material available at 10.1186/s41747-022-00317-6.

## Key points

- Radiomic features automatically extracted from computed tomography were used for artificial intelligence (AI) modelling.
- Four coronavirus disease 2019 (COVID-19) AI classifiers were implemented with different number and type of features.
- These classifiers performed well on both test set and independent dataset.
- Higher performances were associated to models based on radiomic features.
- An automatic pipeline could be implemented for real-time COVID-19 *versus* non-COVID-19 pneumonia classification.

## Background

More than 2 years after the onset of the pandemic, coronavirus disease 2019 (COVID-19) from severe acute respiratory syndrome coronavirus 2 (SARS-CoV-2) still represents a daily reality for many healthcare systems. National vaccination campaigns have greatly reduced the severity and mortality of the disease [1, 2]; however, to date, it is impossible to predict how the virus will evolve, with what consequences, and in what time frame.

Chest computed tomography (CT) remains a relevant tool for COVID-19 diagnosis and management [3], with the most frequent findings including ground-glass opacities, with areas of crazy paving pattern and consolidations in the advanced stages, showing bilateral, peripheral, and basal distribution [4, 5]. This appearance is typical but shows large overlap with the findings of other pulmonary diseases, especially pneumonia from non-COVID-19 viral agents [6]. The differential diagnosis is crucial because the contagiousness of COVID-19 makes it necessary to take actions to prevent the spread of the virus and planning the appropriate clinical strategy.

Given the limits of qualitative pattern assessment, quantitative analysis of CT images has become increasingly important. For example, histogram analysis of HU distribution in CT images allows to calculate quantitative metrics and quantify the extent of pulmonary involvement and the amount of spared, well-aerated lung parenchyma [7]. The interest for radiomics [8], the high-throughput automated extraction and analysis of a large number of quantitative features from medical imaging, has exponentially grown as well, especially with the widespread diffusion of models based on artificial intelligence (AI). Indeed, since the beginning of the pandemic crisis in 2020, these techniques have been largely applied to chest radiographs and CT images of COVID-19 patients to build various predictive classifiers [9–13].

In particular, many authors pointed out the promising role of AI models in differentiating COVID-19 from other types of pneumonia [14–17]. However, most of these models were developed using a limited number of cases or heterogeneous data, for example, including bacterial pneumonias or healthy subjects, which can ease the classification task [18–20]. Moreover, the classifiers focused on viral-only pneumonias were mostly built using single-slice manual segmentations and required also to contour individual lesions. This is not suitable for clinical application since it would need a real-time and automatic analysis, for example, to provide support in case rapid COVID-19 tests are not promptly available (*e.g.*, during night shifts) or yield equivocal results.

Therefore, in this study, we developed a pipeline for quantitative analysis of CT images that included both automatic segmentation and AI-based classifiers to distinguish between COVID-19 and other types of viral (non-COVID-19) pneumonia. We used a large CT dataset to develop multiple AI models implementing different typologies and numbers of features extracted with distinct quantitative approaches. Hence, a comparative performance analysis was performed to select the most promising solution for clinical support.

## Methods

This retrospective study was approved by the local ethics committee. The need for informed consent was waived owing to the retrospective design of the study. All analyses were performed using data of anonymised patients on a workstation with the following characteristics: HP Z8 G4 workstation with a 2.30 GHz processor with 64 cores, 187 GiB memory, and NVIDIA Quadro RTX 6000/8000 graphics card.

### Clinical data and imaging

This study was performed using chest CT images of 1,031 patients with real-time polymerase chain reaction positive for SARS-CoV-2 (647 COVID-19 patients: 458 males, 71%; median age 67 years, interquartile range 23 years) or other types of viral pneumonia (384 non-COVID-19: 236 males, 61%; median age 66 years, interquartile range 20 years), with a CT scan performed within 15 days of serological evidence of infection. All acquisitions were performed in a single hospital with the same patient setup (supine, arms over the head, breath hold) and with the same protocol (unenhanced chest CT) using four different scanners (Brilliance 64, Philips, Amsterdam, the Netherlands; Somatom Definition, Somatom Definition Edge, Somatom Sensation, Siemens Healthineers, Erlangen, Germany).

Even if some differences between CT systems are unavoidable, the equivalence of the acquisition protocols from a dosimetric point of view and the reconstruction algorithms was preliminarily evaluated. For both COVID-19 and non-COVID-19 dataset, more than 90% of CT images were acquired on Siemens scanners and reconstructed with standard kernels for lung parenchyma. The median values (25th, 75th percentiles) of CT dose index (CTDI) for non-COVID-19 and COVID-19 acquisitions were 7.1 (5.8, 8.4) and 6.7 (5.4, 8.7) mGy, respectively. Furthermore, only reconstructed series with slice thickness of 3 mm or lower and with high-resolution reconstruction kernels were considered providing equivalent clinical image quality.

COVID-19 CT images were acquired between March 2020 and March 2021, with 75% of patients belonging to the “second wave” (October to November 2020). A total of 811 patients from the database (496 COVID-19 and 315 non-COVID-19) were randomly chosen for model building, while the others 220 (151 COVID-19 and 69 non-COVID-19, respectively) were used as independent validation set (IVS).

All non-COVID-19 CT images were acquired between January 2015 and October 2019. All information about demographic data, scanner models, and acquisition protocols are reported in Tables S1, S2, and S3 in supplementary materials.

### Quantitative CT pipeline

#### Segmentation of CT images and preprocessing

All CT images were exported from the local picture archiving and communication system and from a research Virtual Network Archive to a dedicated workstation. The images were segmented through the *lungmask* (v0.2.9) [21] Python package, by exploiting the *R231CovidWeb* U-net convolutional network trained with additional COVID-19 data. The accuracy of this tool for automatic segmentation was preliminarily assessed by an experienced radiologist who assigned a 5-point qualitative score to the segmentations obtained from the IVS (220 chest CT scans). The software employed was able to automatically identify the left and right lungs. For each CT images, the lung masks were then divided into 4 geometrical subdivisions (GS) using a dedicated program developed with *JavaScript* macros operating in the *ImageJ* [22] environment. A superior-inferior subdivision was obtained calculating the central slice corresponding to the 50th percentile of total lung volume. A ventral-dorsal subdivision was obtained calculating the line crossing the centroid of left and right lungs in the central slice. Finally, CT images and the corresponding lung masks were resliced into stacks using the *reslice* option within *ImageJ* tool and a bi-cubic interpolation: for quantitative metrics (QM, see section “Quantitative metrics and Gaussian model extension for well-aerated volume estimation of the lung (WAVE)”, the CT slice thickness was set to 3 mm, while for radiomic features (RF, see dedicated section below) an isotropic voxel size of 1.15 mm^3^ was used. This value was selected as it matched with average voxel dimension in CT images with 3 mm of slice thickness.

#### Quantitative metrics and Gaussian model extension for well-aerated volume estimation of the lung (WAVE)

Based on our previous work [7], we calculated the WAVE in two different ways: by integrating histogram data points using fixed threshold in the range ([-950, -700] HU [23–25], WAVE.th) and by applying the Gaussian model (WAVE.f).

In this work, we refined the Gaussian model of the lung from the histogram of CT numbers in segmented images. Briefly, well-aerated lung parenchyma can be described by a Gaussian function fitted over the histogram points around the first peak, which is usually located in a range that goes from -1,000 to -700 HU [26]. WAVE.f is calculated by integrating the Gaussian function over the histogram data points. In this way, in CT images (or its GS) for each histogram, we can define three distributions: “total lung,” “aerated lung,” and “diseased tissues.” The latter is calculated as the difference between HU histogram data points (“total lung”) and Gaussian function representing the “aerated lung.”

A custom software developed in Python3 language [www.python.org] calculates the relative histogram distributions of the HU in the two lungs (2L) and the 4 GS: lower front, upper front, lower dorsal, and upper dorsal. In order to overtake the limitation for the applicability of the Gaussian fit to the histogram data discussed in the previous work, some improvements were implemented.

First, an algorithm for the selection of the histogram data points to be fitted was optimised to assure that the fitted function correctly represents the WAVE.

Subsequently, an iterative loop was implemented: if in one or more lung regions the histogram peak representing the healthy parenchyma was not clearly discernible or when the Gaussian model was not applicable, the results of the Gaussian fit obtained in the other regions were used to extend the model for “aerated lung” where initially failed. Moreover, if the fit was not applicable in the 2L nor in all the GS or the iterative loop failed, the Gaussian function was replaced by histogram data points between -950 and -700 HU (included).

First-order QM (*i.e.*, average HU, percentiles, skewness, and kurtosis) for the “total lung” and “diseased” parenchyma histograms (“ill” QM) were calculated for each CT image in 2L (*n* = 20) and 4 GS (*n* = 80).

Two additional clinical QM were added providing information about the WAVE gradient in the lungs:

$${\displaystyle \begin{array}{c}\frac{Upper}{Lower}=\frac{WAVE.{th}_{UF}\ast Volum{e}_{UF}+ WAVE.{th}_{UD}\ast Volum{e}_{UD}}{WAVE.{th}_{LF}\ast Volum{e}_{LF}+ WAVE.{th}_{LD}\ast Volum{e}_{LD}}\\\\ {}\frac{Front}{Dorsal}=\frac{WAVE.{th}_{UF}\ast Volum{e}_{UF}+ WAVE.{th}_{LF}\ast Volum{e}_{LF}}{WAVE.{th}_{UD}\ast Volum{e}_{UD}+ WAVE.{th}_{LD}\ast Volum{e}_{LD}}\end{array}}$$

where Volume_X_ is the volume of the considered GS, UF is the upper front lung region, UD is the upper dorsal region, LF is the lower front region, and LD is the lower dorsal region. The complete list of QM is reported in supplementary materials, Table S4.

#### Radiomic analysis

RF were extracted in 2L from CT images of the lung using the PyRadiomics Python tool (v3.1) [27]. All RF of the first-order statistics (RF1, *n* = 19) and those of second order (RF2, *n* = 40) derived from the grey-level co-occurrence matrix (GLCM, *n* = 24) and grey-level size zone matrix (GLSZM, *n* = 16) were extracted using a range of HU from -1,020 up to 180 HU. A bin width of 5 HU was chosen for RF1, while three different values were used to extract GLCM (bin width = 5, 25, 50) and GLSZM (bin width = 25, 100, 200) (Table 1).

**Table 1:** Radiomic features extracted

		Number of features	Range (HU)		Bin width	Number of bins	Total number of features
RF1	First-order statistics	19	-1,020	180	5	240	19
RF2	GLCM	24	-1,020	180	5, 25, 50	240, 48, 24	72
	GLSZM	16	-1,020	180	25, 100, 200	48, 12, 16	48

Number of first-order statistics (RF1) and GLCM and GLSZM (RF2) features extracted with PyRadiomics tool together with range of Hounsfield unit (HU), bin width, number of bins used, and the resulting total number of features extracted for each type. *GLCM* Grey-level cooccurrence matrix, *GLSZM* Gray-level size zone matrix, *HU* Hounsfield unit, *RF1* First-order radiomic features, *RF2* Second-order radiomic features

### ML model building for classification of COVID-19 *versus* non-COVID-19

RF1, QM, and RF2 were used to develop four multilayer perceptron (MLP) classifiers, which differ for the numbers and type of metrics used as input (see Table 2), to discriminate images of patients with COVID-19 from non-COVID-19 interstitial pneumonia. The models (type of metrics, total number of features) were the following: *Model1* (RF1 in 2L, *n* = 20), *Model2* (QM in 2L and 4 GS, *n* = 102), *Model3* (RF1 + RF2 in 2L, *n* = 141), and *Model4* (RF1 in 2L + QM in 2L and 4 GS + RF2 in 2L, *n* = 241).

**Table 2:** Main properties and results of each artificial intelligence model

Model	Type of features	Number of radiological features^**a**^	Number of relevant radiological features^**a**^	Test set Accuracy (mean ± standard deviation)	Test set AUC (mean ± standard deviation)
*Model1*	RF1 (2L)	21	10	0.713 ± 0.004	0.768 ± 0.032
*Model2*	QM (2L and 4 GS)	102	26	0.724 ± 0.006	0.800 ± 0.026
*Model3*	RF1 + RF2 (2L)	141	24	0.776 ± 0.003	0.867 ± 0.008
*Model4*	RF1 + QM (2L and 4 GS) + RF2 (2L)	241	32	0.796 ± 0.005	0.870 ± 0.011

Principal characteristics of each model developed. The type of features, the number of initial radiological features, and the final relevant radiological features after LASSO regression used for building each classifier are reported together with results of accuracy and AUC obtained in the test set. Mean and standard deviation values of results were calculated after a 4-fold cross-validation iterated ten times. ^a^Patient age and sex were added as clinical metrics in all models. *AUC* Area under the receiving operating characteristic curve, *2L* Two lungs, *GS* Geometrical subdivisions, *QM* Quantitative metrics, *RF1* First-order radiomic features, *RF2* Second-order radiomic features

In addition, patient age and patient sex were considered as additional features in each model.

All the classifiers were built with *TensorFlow 2.0* (v2.4.0) [www.tensorflow.org] through the following steps, briefly described below, in a pipeline developed in Python language:

1. Least absolute shrinkage and selection operator (LASSO) [28] (Least Absolute Shrinkage and Selection Operator) regression for relevant features selection
2. MLP neural network using early stopping and hyperparameters tune to find the right combination of hyperparameters that maximises the model performance
3. k-fold cross-validation for performance estimation
4. Repetition of k-fold cross-validation

*Scikit-learn* (v0.24.2) Python package [www.scikit-learn.org] was used for the implementation of the LASSO penalised regression algorithm via the LASSO class.

MLP neural networks were built using *binary cross-entropy* as loss function and *Nadam* (stochastic gradient descent with Nesterov momentum) as optimiser. The training/test split was set to be 75/25%, and an early stopping algorithm to avoid overlearning was implemented with a validation split equal to 0.2. An hyperparameter algorithm (*hyperopt*) based on a Bayesian optimisation was used to find the best parameters for the model itself.

After finding the best hyperparameters for the classifiers, we used a k-fold cross-validation (provided by *scikit-learn)* to evaluate the performance of each model: dataset was shuffled making sure that the divisions of the train test were always deterministic and then split into four folds (*k* = 4). The following metrics were used to evaluate the performances of each model: accuracy and area under the receiver-operating characteristic curve (AUC) value. For a more robust assessment of each model, k-fold cross-validation was independently iterated ten times to have a final value of the performance. Average and standard deviation of performance metrics were calculated.

To exclude possible bias due to demographical data or different reconstruction kernels, we implemented three dedicated models: d*emographic-model*, based on all training data using only sex and age and *Model3-B70* and *Model4-B70* based on the same RF and QM of Model3 and Model4, considering only CT images reconstructed with the same B70 kernel (COVID-19, *n* = 237; non-COVID-19, *n* = 285).

The best models were then applied to the IVS based on 220 patients, and specificity and sensitivity were calculated considering COVID-19 as positive and non-COVID-19 as negative. Significance of the differences between ROC curves of the models was tested using the DeLong test.

## Results

### Automatic segmentation

**Fig. 1:**
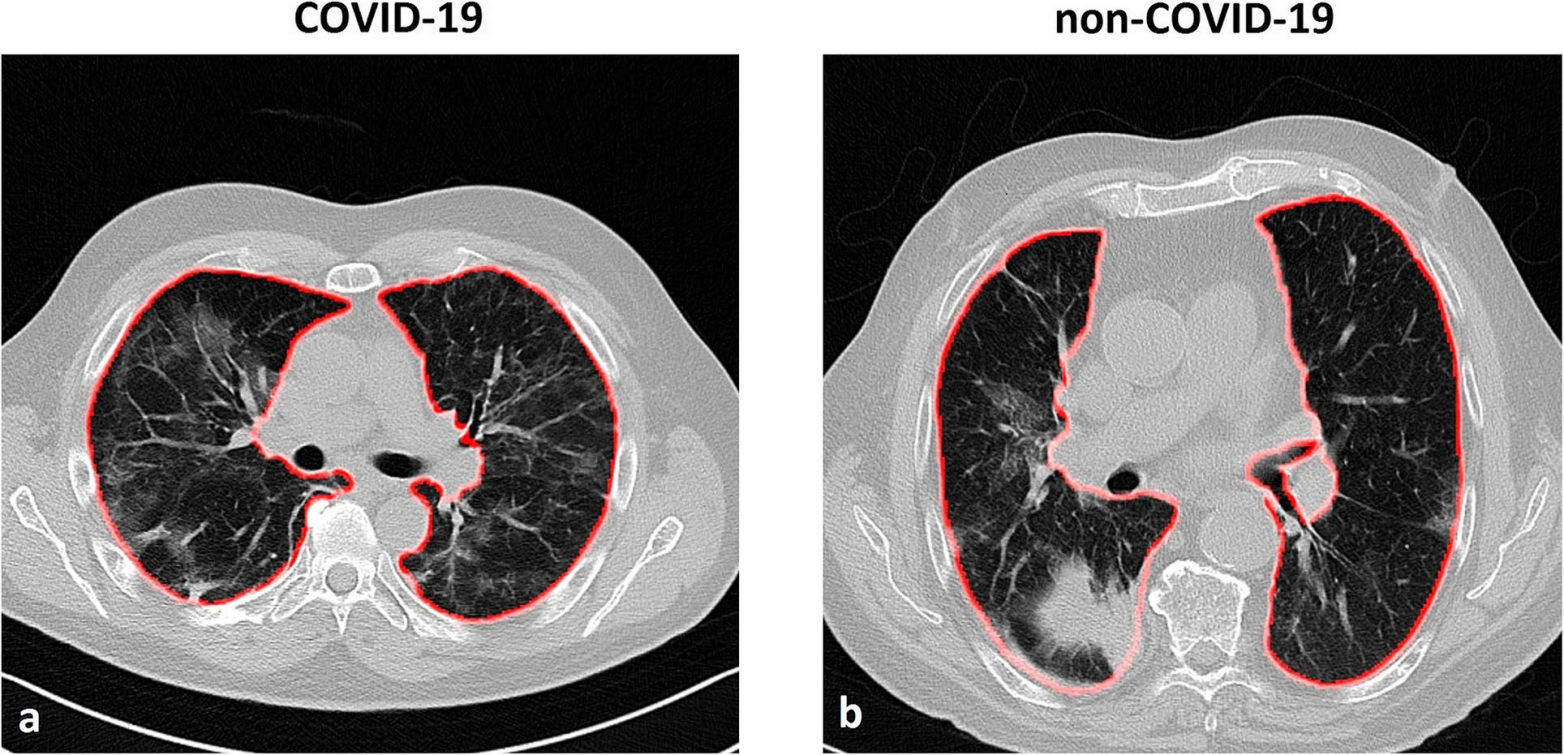
Example of axial slices of computed tomography of patients with COVID-19 (**a**) and another type of non-COVID-19 viral pneumonia, *i.e.*, parainfluenza type 4 virus (**b**). The results of automatic segmentation for the lungs are displayed as a red superimposed contour

The quality of segmentation obtained with this automatic tool resulted excellent (score = 5, *i.e.*, segmentation corresponding to the ideal result for the reader) in 194/220 (88%) of the cases or good (score = 4, *i.e.*, segmentation with small imperfection negligible for the reader) in 15/220 (7%) of the cases. Even if some limited inaccuracies were detected (11/220, 5%), no manual corrections were performed because the effects on subsequent analysis were considered irrelevant, according to our previous study [29]. An example of the automatic segmentation for COVID-19 and non-COVID-19 CT images is reported in Fig. 1.

### Relevant features

The number of relevant features for each model after LASSO regression is reported in Table 2 (the complete list of relevant metrics is reported in supplementary materials, Table S5).

The models were implemented with an increasing complexity of number and typology of features. The LASSO reduced the numbers of redundant features: the higher was the radiological initial features, the higher was the resulting reduction due to the presence of more correlated features.

### Classifier performances

**Fig. 2:**
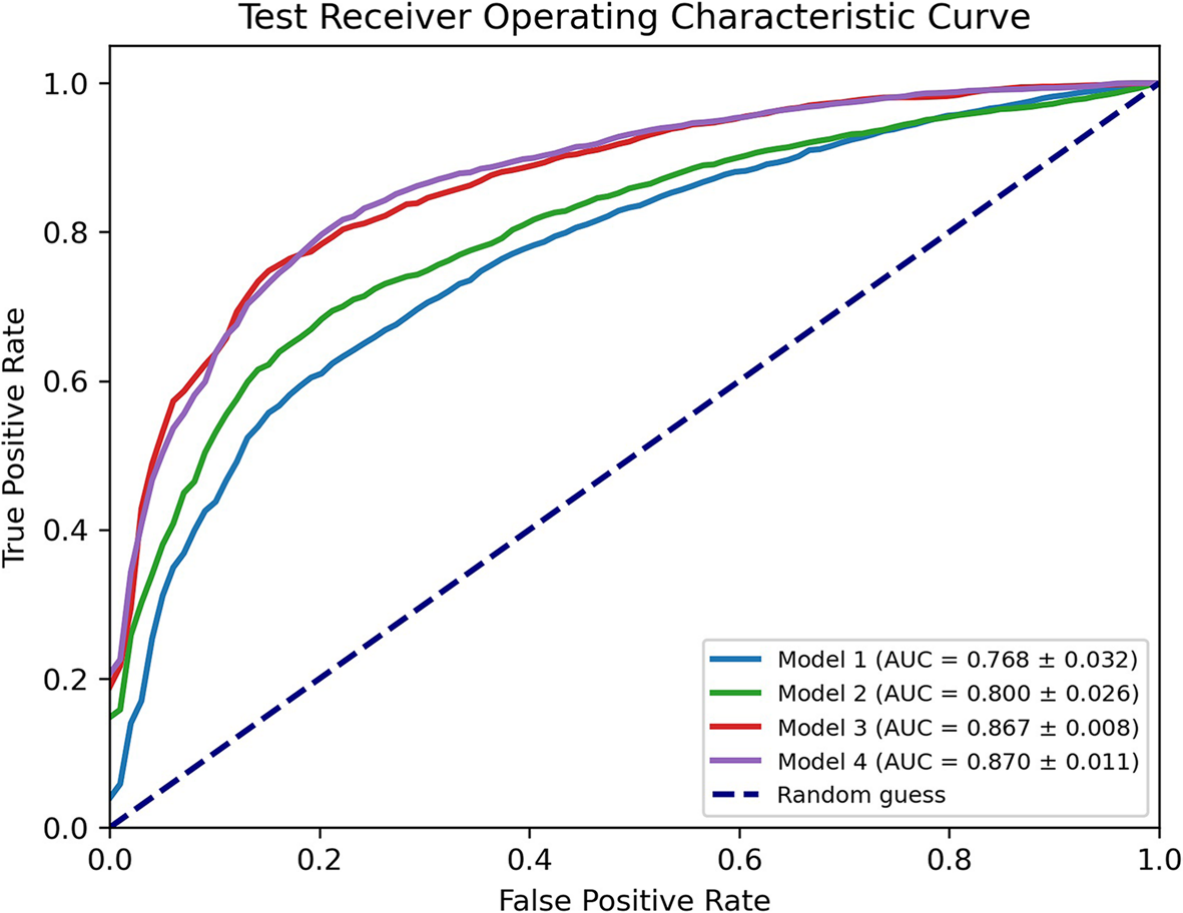
Receiving operating characteristic (ROC) curve of all models for classification of viral pneumonia (COVID-19 *versus* non-COVID-19) on the test set (811 computed tomography images). For each model, the mean ROC curve obtained with fourfold cross-validation iterated ten times is represented. The mean and standard deviation of the area under the curve (AUC) values are reported

The performances of four MLP models on the test set (811 patients) are shown in Fig. 2. *Model1,* based on only ten relevant RF1features*,* showed poorer performance when compared to the others, especially as AUC values (0.768 ± 0.032, mean ± standard deviation). An increase in accuracy was observed in *Model2* by adding the QM based on the Gaussian model of the lung calculated in 2L and the 4 GS (AUC = 0.800 ± 0.026). On the other hand, a greater increase in performances was observed by adding second-order features to *Model1* (*i.e.*, *Model3* = *Model1* + RF2), with an AUC of 0.867 ± 0.008. The highest number of relevant radiological features was associated with *Model4*; they were a subset of those from *Model3* and *Model4*.

**Fig. 3:**
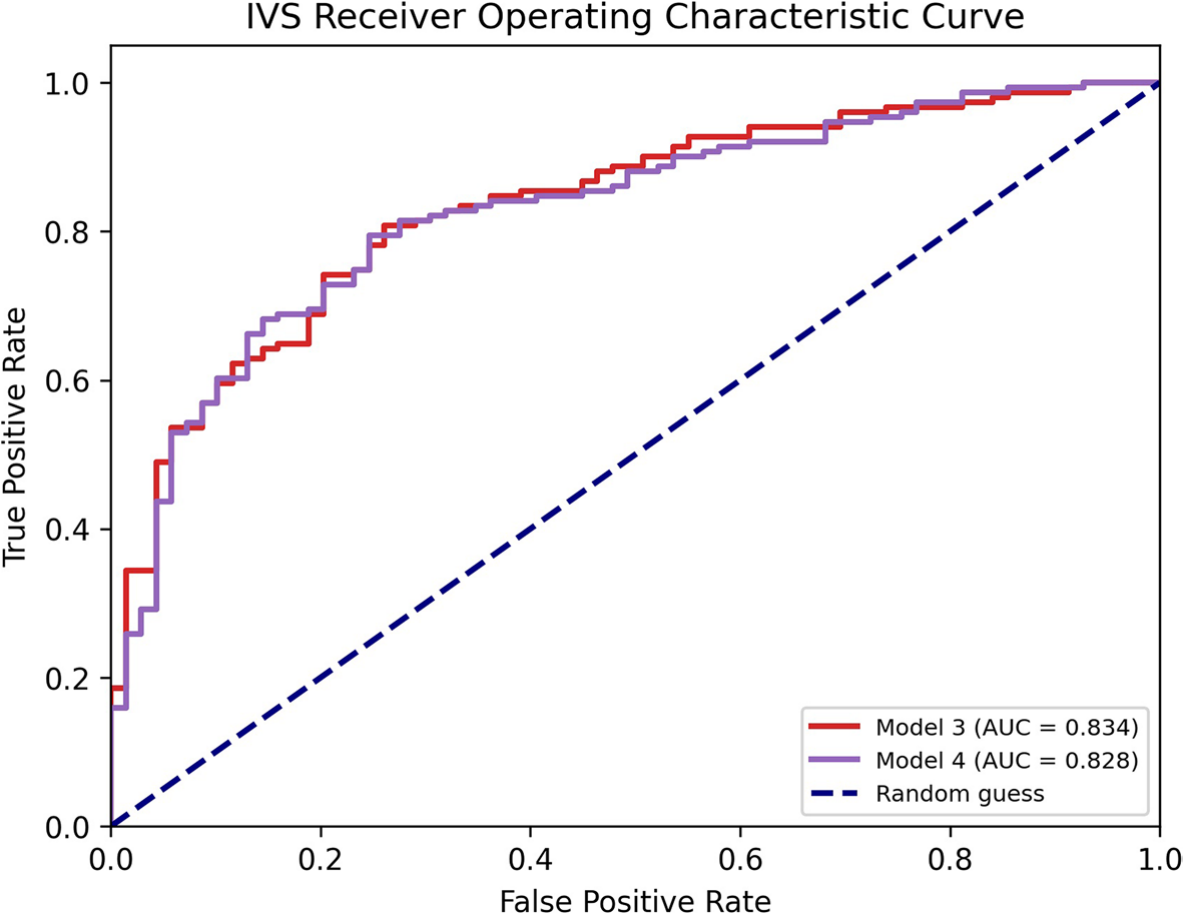
Receiving operating characteristic (ROC) curve of *Model3*, *Model4, demographic model*, *Model3-B70*, and *Model4-B70*. For each model, the mean ROC curve obtained with fourfold cross-validation iterated ten times is represented. The mean ± standard deviation of the area under the curve (AUC) values are reported

Figure 3 shows the AUC curves of *demographic model, Model3* and *Model3-B70, and Model4 and Model4-B70*. It is evident that demographic data alone are not sufficient to obtain a good classification (*AUC* = 0.54). On the other hand, both models built with a subset of CT images with the same kernel (*Model3-B70* and Model4-B70) were not significantly different (*p* > 0.071, DeLong test) from the original models based on the entire training set (*Model3* and *Model4*).

Since *Model3* and *Model4* showed the higher performances on test set, they were both applied to the IVS to validate their consistency in the classification task.

**Fig. 4:**
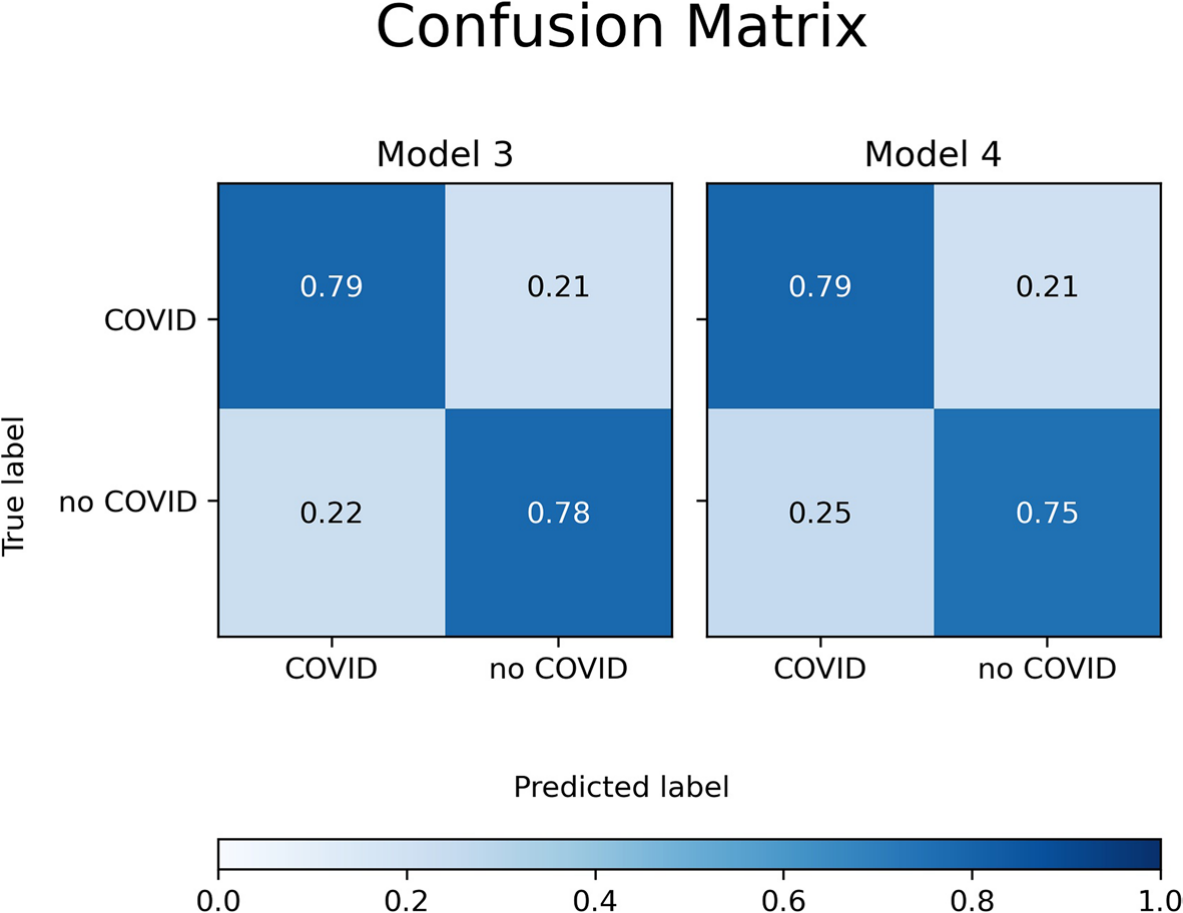
Receiving operating characteristic (ROC) curve and area under the curve (AUC) values of the two best models (*Model3* and *Model4*) applied on the independent validation set. *IVS* Independent validation set

The results of the application of AI models to the IVS (220 CT images) are shown in Table 3 and in Fig. 4. In both cases, AUC values moved from 0.87 (test set) to 0.83 (IVS). The differences between ROC curves of these two models were not significative (*p* = 0.896, DeLong test).

**Table 3:** Performances of Model3 and Model4 on the independent validation set

	Sensitivity	Specificity	Accuracy	AUC
***Model3***	0.788	0.783	0.786	0.834
***Model4***	0.788	0.754	0.777	0.828

Performance metrics obtained for the application of the two best models (*Model3* and *Model4*) to the independent validation set (220 CT images). In the table are reported sensitivity, specificity, accuracy, and AUC. A threshold of 50% to the predicted value was applied to discriminated COVID and non-COVID patients. *AUC* area under the receiving operating characteristic curve

**Fig. 5:**
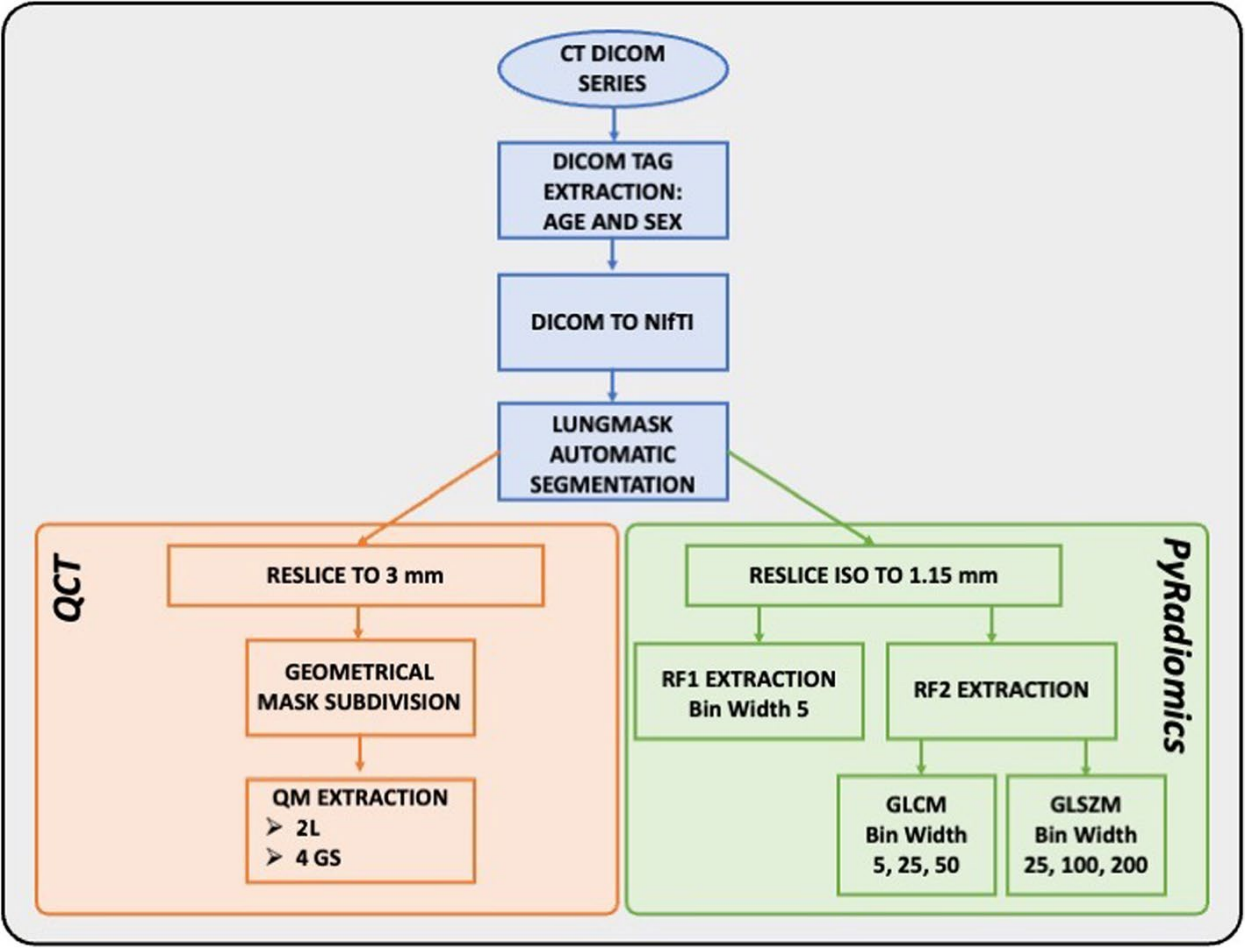
Confusion matrices of the two best models (*Model3* and *Model4*) applied to the independent validation set (220 patients)

A common property of the two models was that specificity and sensitivity were greater than 0.75 in all cases, as can be seen in the diagonals of the confusion matrices shown in Fig. 5.

## Discussion

The goal of this work is to develop AI models for classification of CT images of patients with different types of viral pneumonia to support clinical decisions.

For this purpose, an automated pipeline for quantitative analysis and extraction of radiomic features from CT images of the chest was implemented and optimised.

The interest in developing AI models applied to COVID-19 is growing worldwide [30]. For instance, Harmon et al. [31] and Jin et al. [32] proposed deep learning-based algorithms for COVID-19 detection on CT images. In both studies, the classification task was between a sample of patients positive for SARS-CoV-2 infection and a control group composed of patients with various other clinical conditions including cancer staging, emergency care, and pneumonias from bacteria or influenza, with reported AUC values greater than 0.90.

In a more challenging scenario, Bai et al. [18] described the performance of human readers in distinguishing different types of viral pneumonia, with an accuracy ranging between 60 and 83% [18]. In this study, a high specificity (up to 1.00) but a moderate sensitivity as low as 0.67 were reported.

This suggests the need for a reliable and rapid classification tool able to provide a prompt response in front of patients with suspected respiratory infection. In this sense, an automated, “real-time” AI system could identify COVID-19 patients early on, allowing appropriate precautions and clinical decision to be taken even if the laboratory test is not available and widening the clinical view of the disease condition.

Wang et al. [17, 33] proposed a method for the classification of different types of viral pneumonia and COVID-19 based on radiomics features extracted from the lung and lesion manually segmented on CT images. In these studies, higher values of AUC are linked to the models built with lesion segmentations (AUC = 0.87). Despite the promising results, the single slice manual segmentation is a time-consuming approach unfeasible in the everyday clinical practice and does not allow a predictive proposal of disease in real time.

Cardobi et al. [34] extracted RF using PyRadiomics tool from automatic lung segmentations and developed a model to distinguish CT images of COVID-19 patients from other interstitial pneumonias with AUC values of 0.77. However, the limited number of CT scans employed in this work (*n* = 115) and the lack of independent validation raise some caveats to its clinical implementation.

We developed four AI models based on different sets of QM and RF with AUC values ranging from 0.77 to 0.87.

The four models were developed by considering different approaches with increasing complexity and number of features. As expected, the greater the number of radiological features, the higher the results in terms of specificity, sensitivity, accuracy, and AUC.

Although age and sex were found to be relevant features following LASSO regression in all models, adding imaging features to the models significantly improved the AUC value.

The best models were associated to a higher number of extracted features, *i.e.*, *Model3*, based on 24 useful/independent features selected with LASSO optimisation from 141 different initial metrics, and *Model4*, based on 32 useful/independent features selected with LASSO optimisation from a set of 241 different metrics. Even if PyRadiomics tool allows to extract several classes of second-order features, differently from other works [10, 11, 17, 32, 33], we selected a priori the classes we thought were more suitable for our classification task, *i.e.*, GLCM and GLSZM. In fact, GLCM class includes the most commonly implemented features across the radiomic applications of texture analysis: as they describe the voxel relationships within an image, they could help to distinguish between different lung parenchymal patterns [35–37]. On the other hand, the GLSZM and its derived features describe areas with uniform grey levels in terms of size and intensity, which could be able to identify different types of lung lesions between viral pneumonias.

Furthermore, in the absence of reference values for second-order features extraction, three different combinations of bin were used. The choice was based on the HU distribution of viral pneumonia as a compromise between the noise contribution and the loss of texture information [38]. Indeed, different features were relevant with different bin number used for extraction (Table S5 supplementary materials).

The equivalence of models based on all the training set with those based on homogeneous images, *i.e.*, reconstructed with the same kernel (Fig. 3), may have multiple explanations. First, the reconstruction kernel used, although nominally different, all belong to the same class for the evaluation of lung parenchyma. Second, RF2 that can be highly dependent on the reconstruction kernel and image noise were extracted after an isotropic rescaling. This operation, applied on a volume as large as the entire lung parenchyma, reduces the noise in the images without affecting the signal that RF2 can detect.

We also considered a geometric classification by dividing the lung volume into 4 GS because it is well reported that COVID-19 lesions show predominant involvement of the lower and dorsal portion of the lung [39]. In *Model4*, obtained from all types of RF and QM, the relevant features were found to be a subset of both QM (in 2L and 4 GS) and RF, showing the usefulness of different methodological approach of metrics calculation.

Independent validation results provided equivalent performances between *Model3* and *Model4* (AUC of 0.833 and 0.828, respectively; *p* = 0.896). *Model3* requires a simple isotropic voxel reslice operation and is based on radiomic features that can be extracted with an open-source package; however, they do not have a clear biological correlate, so they cannot be directly interpreted. On the contrary, *Model4* requires more elaborate preprocessing for the geometric partitioning of masks and an independent code for applying the Gaussian model of the lung, but it allows to calculate clinically interpretable quantitative metrics. As shown, the two models differ in the applicability of the methods and the interpretability of the results. For implementation in clinical practice, we consider the applicability of a model more important than the interpretability of its features, as well as the smaller number of included features which reduces the risk of overfitting. However, the present work only aimed to build an optimised pipeline for automatic segmentation and quantitative analysis of chest CT, comparing different possible approaches for the AI modelling. For a more appropriate interpretation of the classifier performance, future developments should involve comparison with the performance of human readers and decision curve analysis [40] to verify whether *Model3* actually provides added value in supporting clinical practice.

**Fig. 6:**
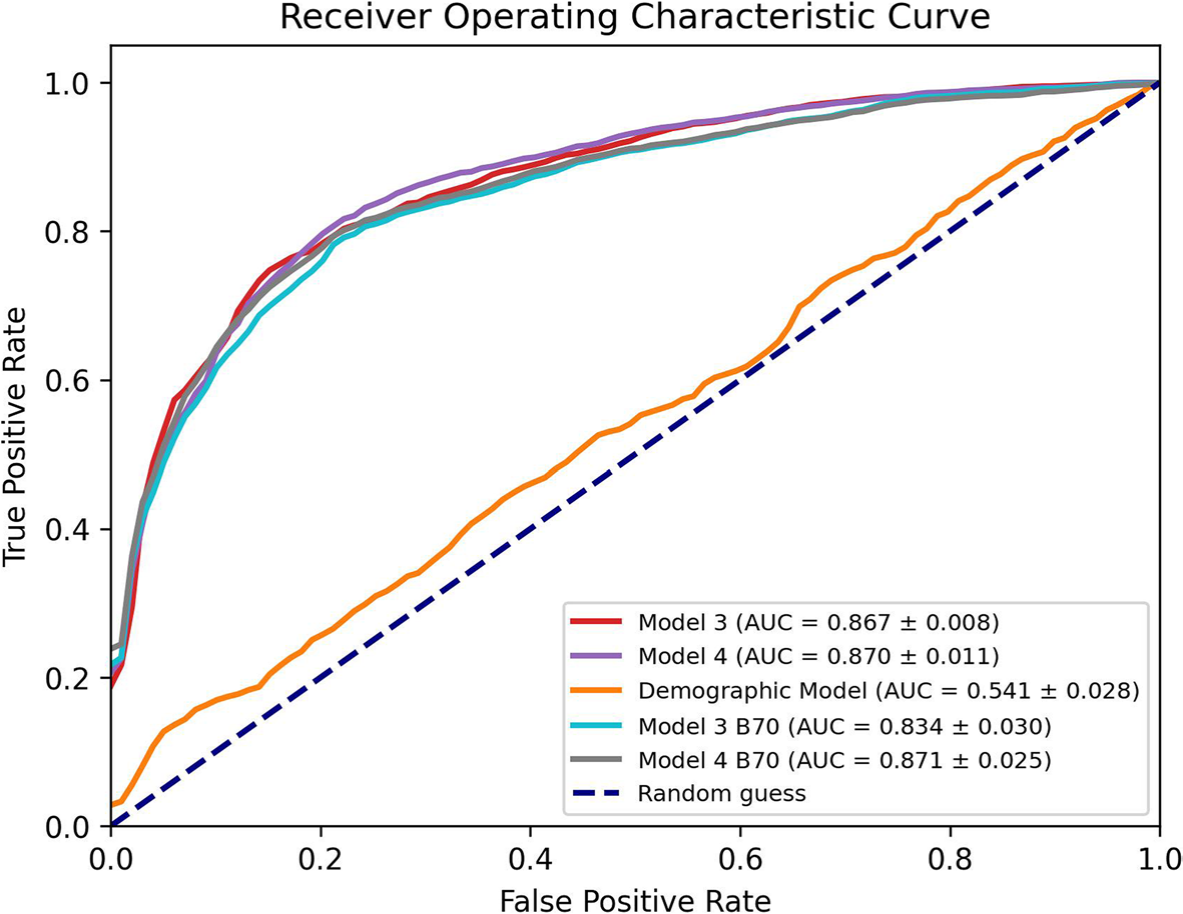
Schematic representation of pipeline used to calculate quantitative metrics and radiomic features in computed tomography images

Notably, in this work, we used a segmentation algorithm whose result proved consistent with the task of our interest. The application of the entire pipeline developed in this work, which also includes automatic segmentation, has led to promising results in terms of diagnostic accuracy without any human intervention. In addition, the time for running the entire pipeline (Fig. 6) for a single CT series is less than 1 min using the workstation.

This work also has some limitations. The model was developed by using COVID-19 CT scans acquired between March 2020 and March 2021; any radiological differences ascribable to different and successive variants of SARS-CoV-2 may not be properly accounted for. However, given the protective role of SARS-CoV-2 vaccination [41, 42], it can be assumed that CT scan will be increasingly reserved to patients with moderate-to-severe lung involvement who are more likely to show the “typical” bilateral parenchymal infiltrates, in overlap with other viral pneumonias.

Another limitation lies in the inclusion of chest CT scans within 15 days from molecular evidence of SARS-CoV-2 infection; therefore, it is possible that some of the selected patients had mixed pneumonia, even though the large dataset used should have minimised the impact of this occurrence. Finally, although validation with independent data was performed, more consistent results would be obtained with the use of CT images from other hospitals. Moreover, the number of images used for training is not equally distributed between the two categories; thus, the scenario assumed here is of a higher prevalence of COVID-19 cases compared to the other types of viral pneumonia. If epidemiological conditions would change significantly, this imbalance could constitute a bias.

In conclusion, four MLP classifiers have been implemented in Python language to classify patients with COVID-19 and non-COVID-19 viral pneumonia using QM and features extracted from chest CT images. The results showed that all classifiers performed well on the test set, indicating overall good performances in the diagnostic task. When applied to independent validation set, the two best models provided good performances and equivalent results despite the differences of the interpretability of their metrics and of the applicability of their methods.

## Supplementary Information

**Additional file 1: Table S1.** Demographic data of patients, scanner models and acquisition parameters of all CT used in this work. **Table S2.** Demographic data of patients, scanner models and acquisition parameters of CT used for the training set. **Table S3.** Demographic data of patients, scanner models and acquisition parameters of CT used for the independent validation set (IVS). **Table S4.** List and number of Quantitative Metrics extracted using gaussian model applied both on bilateral lungs and 4 geometrical subdivisions. **Table S5.** Number and typology of relevant features both of first order (RF1) and second order (RF2: GLCM and GLSZM) for each model after LASSO regression. Legend: UF = Upper Front, UD = Upper Dorsal, LF = Lower Front, LD = Lower Dorsal.

## Data Availability

The datasets generated and/or analysed during the current study are not publicly available because of the terms of the research but are available from the corresponding author on reasonable request.

## Abbreviations

2L: Two lungs
AI: Artificial intelligence
AUC: Area under the receiving operating characteristic curve
CT: Computed tomography
GS: Geometrical subdivisions
IVS: Independent validation set
LASSO: Least absolute shrinkage and selection operator
MLP: Multilayer perceptron
QM: Quantitative metrics
RF: Radiomic features
WAVE: Well-aerated volume estimation
