# Supplementary material for: Artificial intelligence for differentiating COVID-19 from other viral pneumonias on CT: comparative analysis of different models based on quantitative and radiomic approaches
Source: Eur Radiol Exp. 2023 Jan 24;7:3. doi: 10.1186/s41747-022-00317-6 (PMC9870776; doi:10.1186/s41747-022-00317-6)
Supplement: Supplementary file 1 — Additional file 1: Table S1. Demographic data of patients, scanner models and acquisition parameters of all CT used in this work. Table S2. Demographic data of patients, scanner models and acquisition parameters of CT used for the training set. Table S3. Demographic data of patients, scanner models and acquisition parameters of CT used for the independent validation set (IVS). Table S4. List and number of Quantitative Metrics extracted using gaussian model applied both on bilateral lungs and 4 geometrical subdivisions. Table S5. Number and typology of relevant features both of first order (RF1) and second order (RF2: GLCM and GLSZM) for each model after LASSO regression. Legend: UF = Upper Front, UD = Upper Dorsal, LF = Lower Front, LD = Lower Dorsal. [file 41747_2022_317_MOESM1_ESM.pdf]

ELECTRONIC SUPPLEMENTARY MATERIALS

Artificial intelligence for differentiating COVID-19 from other viral pneumonias on CT: comparative analysis of different models based on quantitative and radiomic approaches

	All Data COVID				All Data non-COVID			
	**Age (years)**		**Sex**	**n**	**Age (years)**		**Sex**	**n**
**Demographic Data**	25th percentile	55	M	458	25th percentile	54	F	148
	Median	67	F	189	Median	66	M	236
	75th percentile	78	F/M	41%	75th percentile	74	F/M	63%
	IQR	23		647	IQR	20		384
	**Scanner model**	**n**	**Kernel**	**n**	**Scanner model**	**n**	**Kernel**	**n**
**Siemens**	Definition	273	B30f	1	Definition	133	B30f	0
	Sensation64	63	B31f	3	Sensation64	217	B31f	0
	DefinitionEdge	311	Bl57-1	287	DefinitionEdge	5	Bl57-1	2
			Bl57-3	24			Bl57-3	3
			B70f	332			B70f	349
	**Tot Siemens:**	**647**	B80f	0	**Tot Siemens:**	**355**	B80f	1
**Philips**	Brilliance64	0	D	0	Brilliance64	29	D	1
			L	0			L	1
			YA	0			YA	0
			YB	0			YB	21
	**Tot Philips:**	**0**	YC	0	**Tot Philips:**	**29**	YC	6
**kV_p and slice Thickness**	**kVp**	**n**	**Sl Thk (mm)**	**n**	**kVp**	**n**	**Sl Thk (mm)**	**n**
	80	1	1	44	80	10	1	102
	100	125	3	603	100	93	3	279
	120	446	Other	0	120	270	Other	3
	140	75			140	11		

Table S1 Demographic data of patients, scanner models and acquisition parameters of all CT used in this work.

	Training Set COVID				Training Set non-COVID			
	Age (years)		Sex	n	Age (years)		Sex	n
Demographic Data	25th percentile	55	M	347	25th percentile	53.5	M	188
	Median	67	F	149	Median	65	F	127
	75th percentile	78	F/M	43%	75th percentile	74	F/M	68%
	IQR	23	n:	496	IQR	20.5	n:	315
	model		Kernel	n	model		Kernel	n
Siemens	Definition	197	B30f	1	Definition	112	B30f	0
	Sensation64	44	B31f	3	Sensation64	174	B31f	0
	DefinitionEdge	255	Bl57-1	254	DefinitionEdge	0	Bl57-1	0
			Bl57-3	1			Bl57-3	0
			B70f	237			B70f	285
	**Tot Siemens**	**496**	B80f	0	**Tot Siemens**	**286**	B80f	1
Philips	Brilliance64	0	D	0	Brilliance64	29	D	1
			L	0			L	1
			YA	0			YA	0
			YB	0			YB	21
	**Tot Philips**	**0**	YC	0	**Tot Philips**	**29**	YC	6
kV_p and slice Thickness	**kVp**		**Sl Thk (mm)**	**n**	**kVp**		**Sl Thk (mm)**	**n**
	80	0	1	17	80	8	1	89
	100	99	3	479	100	82	3	224
	120	340	Other	0	120	216	Other	2
	140	57			140	9		

Table S2 Demographic data of patients, scanner models and acquisition parameters of CT used for the training set.

	IVS COVID				IVS nonCOVID			
	**Age (years)**		**Sex**	**n**	**Age (years)**		**Sex**	**n**
**Demographic Data**	25th percentile	58	M	111	25th percentile	60	M	48
	Median	67	F	40	Median	68	F	21
	75th percentile	79	F/M	36%	75th percentile	75	F/M	44%
	IQR	20		151	IQR	15		69
	**model**		**Kernel**	**n**	**model**		**Kernel**	**n**
**Siemens**	Definition	76	B30f	0	Definition	21	B30f	0
	Sensation64	19	B31f	0	Sensation64	43	B31f	0
	DefinitionEdge	56	Bl57-1	33	DefinitionEdge	5	Bl57-1	2
			Bl57-3	23			Bl57-3	3
			B70f	95			B70f	64
	**Tot Siemens**	**151**	B80f	0	**Tot Siemens**	**69**	B80f	0
**Philips**	Brilliance64	0	D	0	Brilliance64	0	D	0
			L	0			L	0
			YA	0			YA	0
			YB	0			YB	0
	**Tot Philips**	**0**	YC	0	**Tot Philips**	**0**	YC	0
**kV_p and slice Thickness**	**kVp**	**n**	**Sl Thk (mm)**	**n**	**kVp**		**Sl Thk (mm)**	**n**
	80	1	1	27	80	2	1	13
	100	26	3	124	100	11	3	55
	120	106	Other	0	120	54	Other	1
	140	18			140	2		

Table S3 Demographic data of patients, scanner models and acquisition parameters of CT used for the independent validation set (IVS).

Quantitative Metrics (QM)	Number of QM
Mean HU, p25th, p50th , p75th ,p90th , Standard Deviation, Skewness, Kurtosis, mu.fit, sigma.fit WAVE.fit, WAVE.th, Mean HU ill, Standard Deviation ill, p25th ill, p50th ill, p75th ill, p90th ill, Skewness ill, Kurtosis ill	20

Table S4 List and number of Quantitative Metrics extracted using gaussian model applied both on bilateral lungs and 4 geometrical subdivisions.

Model	Relevant features	Number of relevant features
Model 1	Patient Age, Patient Sex, 10 percentile, Interquartile Range, Minimum, Range, Skewness, Total Energy, Uniformity, Variance	10
Model 2	Patient Age, Patient Sex, Skewness BL, p75th BL, Standard Deviation ill BL, Skewness ill BL, Kurtosis LF, p75th LF, Standard Deviation ill LF, p50th ill LF, p75th ill LF, p75th UF, p90th ill UF, skewness ill UF, Kurtosis LD, p90th LD, Standard Deviation ill LD, skewness ill LD, Kurtosis ill LD,p50th UD, Skewness UD, Kurtosis UD, p90th UD, Standard Deviation ill UD, Upper/Lower, Front/Dorsal	26
Model 3	Patient Age, Patient Sex, 10 Percentile, Minimum, Range, Skewness, Variance, Difference Variance, Joint Entropy, Imc2 25, Inverse variance 25, Cluster Prominence 50, Cluster Shade 50, Gray Level Non Uniformity 25, Gray Level Non Uniformity Normalized 25, Low Gray Level Zone Emphasis 25, Zone Percentage 25, Large Area High Gray Level Emphasis 100, Size Zone Non Uniformity Normalized 100, Small Area Low Gray Level Emphasis 100, Zone Percentage 100, Large Area High Gray Level Emphasis 200, Small Area High Gray Level Emphasis 200, Zone Percentage 200	24
Model 4	Patient Age, Patient Sex, Standard Deviation ill BL, Skewness ill BL, Kurtosis LF, p90th UF, Standard Deviation ill LF, Kurtosis UF, p90th ill UF, Skewness LD, p75th ill LD, Skewness ill LD, Kurtosis ill LD, Skewness UD, Kurtosis ill UD, Front/Dorsal, Minimum, Joint Entropy, Maximum Probability 5, Cluster Shade 25, Imc2 25, Inverse Variance 25, Cluster Shade 50, Imc 2 50, Gray Level Non Uniformity 25, Gray Level Non Uniformity Normalized 25, Low Gray Level Zone Emphasis 25, Large Area High Gray Level Emphasis 100, Small Area Low Gray Level Emphasis 100, Size Zone Non-Uniformity 200, Small Area High Gray Level Emphasis 200, Zone Percentage 200	32

Table S5 Number and typology of relevant features both of first order (RF1) and second order (RF2: GLCM and GLSZM) for each model after LASSO regression. Legend: UF = Upper Front, UD = Upper Dorsal, LF = Lower Front, LD = Lower Dorsal.

The radiomic quality score (RQS) was calculated using the platform available at https://www.radiomics.world/rqs . In this work RQS=14 was calculated.
